# Diagnostic Accuracy of Lateral Flow Urine LAM Assay for TB Screening of Adults with Advanced Immunosuppression Attending Routine HIV Care in South Africa

**DOI:** 10.1371/journal.pone.0156866

**Published:** 2016-06-07

**Authors:** Yasmeen Hanifa, Katherine L. Fielding, Violet N. Chihota, Lungiswa Adonis, Salome Charalambous, Alan Karstaedt, Kerrigan McCarthy, Mark P. Nicol, Nontobeko T. Ndlovu, Faieza Sahid, Gavin J. Churchyard, Alison D. Grant

**Affiliations:** 1 London School of Hygiene & Tropical Medicine, London, United Kingdom; 2 Aurum Institute, Johannesburg, South Africa; 3 University of the Witwatersrand, Johannesburg, South Africa; 4 Mamelodi Hospital, Pretoria, South Africa; 5 Department of Medicine, Chris Hani Baragwanath Hospital, Johannesburg, South Africa; 6 National Health Laboratory Service, Johannesburg, South Africa; 7 University of Cape Town, Cape Town, South Africa; 8 Advancing Care and Treatment (ACT) for TB/HIV, South African Medical Research Council Collaborating Centre for HIV and TB, Cape Town, South Africa; Médecins Sans Frontières (MSF), INDIA

## Abstract

**Background:**

We assessed the diagnostic accuracy of Determine TB-LAM (LF-LAM) to screen for tuberculosis among ambulatory adults established in HIV care in South Africa.

**Methods:**

A systematic sample of adults attending for HIV care, regardless of symptomatology, were enrolled in the XPHACTOR study, which tested a novel algorithm for prioritising investigation with Xpert MTB/RIF. In this substudy, restricted to participants with enrolment CD4<200x10^6^/l, urine was stored at enrolment for later testing with LF-LAM. Sputum was sent for immediate Xpert MTB/RIF if any of: current cough, fever ≥3 weeks, body mass index (BMI)<18.5kg/m^2^, CD4<100x10^6^/l (or <200x10^6^/l if pre-ART), weight loss ≥10% or strong clinical suspicion were present; otherwise, sputum was stored for Xpert testing at study completion. Participants were reviewed monthly, with reinvestigation if indicated, to 3 months, when sputum and blood were taken for mycobacterial culture. We defined tuberculosis as “confirmed” if Xpert, line probe assay or culture for *M*. *tuberculosis* within six months of enrolment were positive, and “clinical” if tuberculosis treatment started without microbiological confirmation.

**Results:**

Amongst 424 participants, 61% were female and 57% were taking ART (median duration 22 months); median age, CD4 and BMI were 39 years, 111x10^6^/l, and 23 kg/m^2^. 56/424 (13%) participants had tuberculosis (40 confirmed, 16 clinical). 24/424 (5.7%) vs. 8/424 (1.9%) were LAM-positive using grade 1 vs. grade 2 cut-off. Using grade 1 cut-off, sensitivity for confirmed TB (all clinical TB excluded) was 12.5% (95% CI 4.2%, 26.8%) and in CD4<100x10^6^/l vs. CD4 ≥100x10^6^/l was 16.7% (95% CI 4.7%, 37.4%) vs. 6.3% (95% CI 0.2%, 30.2%). Specificity was >95% irrespective of diagnostic reference standard, CD4 stratum, or whether grade 1 or grade 2 cut-off was used.

**Conclusion:**

Sensitivity of LF-LAM is too low to recommend as part of intensified case finding in ambulatory patients established in HIV care.

## Introduction

The global HIV-associated tuberculosis (TB) epidemic remains a huge public health challenge, with sub-Saharan Africa accounting for the vast majority of HIV-positive individuals diagnosed with and dying from TB. [1] Diagnosis of TB in people living with HIV (PLHIV) is complicated by limitations of available diagnostics and the effect of immunosuppression on clinical presentation of TB, e.g. reliance on sputum samples, the high proportion with smear-negative or extrapulmonary disease, [2] and slow turnaround time for mycobacterial culture. The World Health Organization recommends, as part of activities to address HIV-related TB, regular screening for active TB of all PLHIV followed by Xpert MTB/RIF (Cepheid, Sunnyvale, CA) as the primary diagnostic test. [3] Xpert MTB/RIF has far greater sensitivity than smear and provides results in under two hours, but like mycobacterial culture is expensive and laboratory-based, which presents challenges for resource-limited settings. [3]

Testing for lipoarabinomannan (LAM), a cell wall lipopolysaccharide specific to mycobacteria that is detectable in urine, is attractive as a screening tool for PLHIV, given a low-cost point-of-care lateral-flow LAM assay (LF-LAM) (Determine TB-LAM; Alere, USA), potential for rapid TB diagnosis, low biosafety risk, and ease of sample collection. Evaluations of LF-LAM as a screening tool for TB have been undertaken in ambulatory patients in Ethiopia and South Africa either prior to antiretroviral therapy (ART) initiation, [4, 5] or on receiving a positive HIV diagnosis at HIV counselling and testing services (HCT). [6, 7] In these groups LF-LAM sensitivity, compared to bacteriologically-confirmed TB, was inadequate as a stand-alone test, though improved at lower CD4 cell counts. When evaluated amongst hospitalised HIV-positive patients with TB symptoms in Uganda and South Africa sensitivity was much greater, particularly amongst those with advanced immunosuppression, suggesting utility as a rule-in test in this population. [8–10]

There are no published studies, to our knowledge, evaluating LF-LAM as a screening tool for TB as part of intensified case finding for ambulatory patients established in HIV care (rather than at their initial assessment). The aim of our study was to evaluate the diagnostic accuracy of LF-LAM among adults with advanced immunosuppression (CD4 <200x10^6^/l) established in HIV care. Our study contributed data to a systematic review of LF-LAM for the diagnosis and screening of active TB in PLHIV, which informed the recently published World Health Organization (WHO) policy guidance.[11]

## Methods

**Fig 1:**
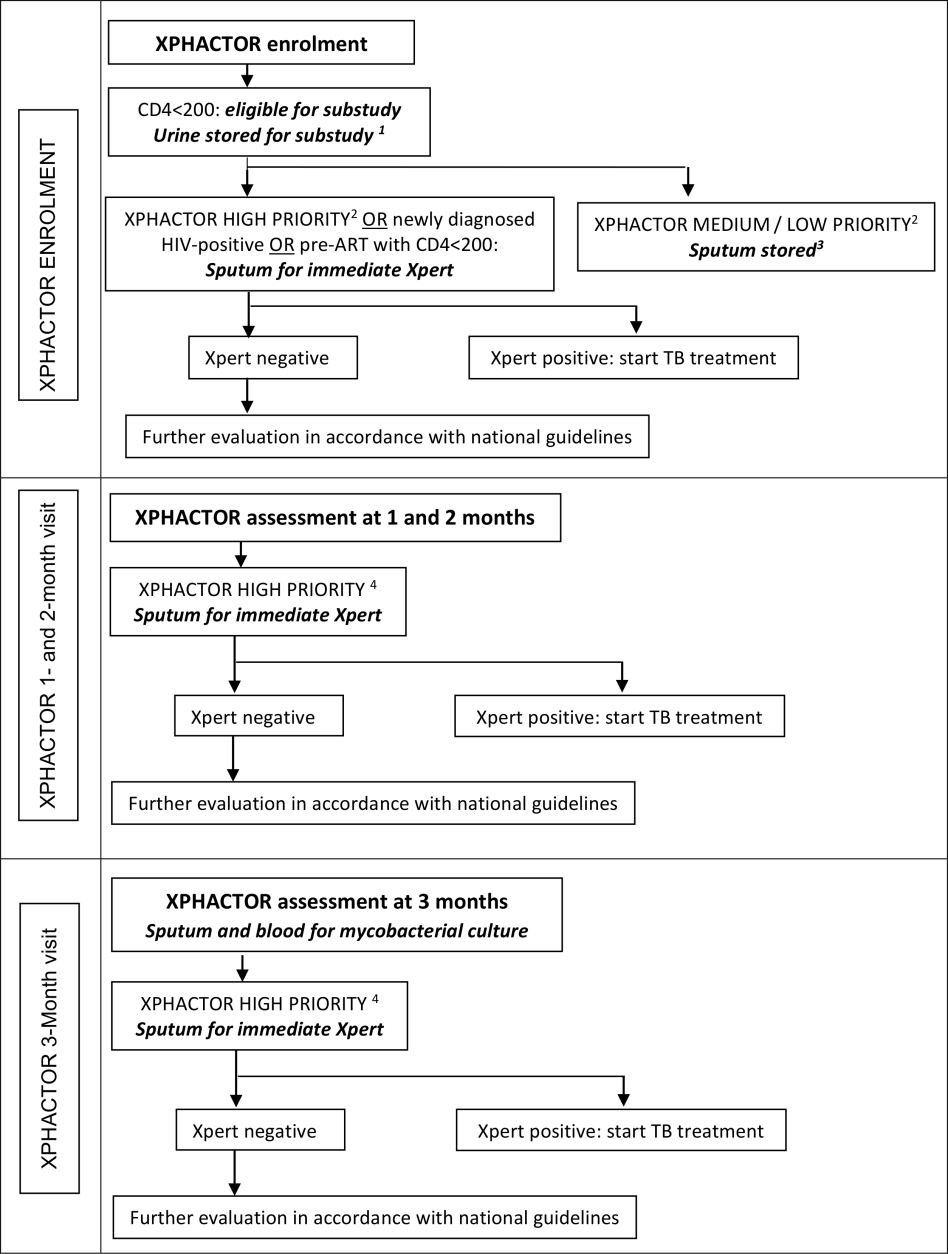
XPHACTOR study flow and entry point to the LAM substudy. ^1^ Samples tested with LF-LAM at the end of the study. ^2^ High priority (any of: current cough, fever ≥ 3 weeks, body mass index (BMI) <18.5 kg/m^2^, CD4 <100x10^6^/l, measured weight loss ≥10% in preceding 6 months, or other feature raising high clinical suspicion of TB); medium priority (any of: fever < 3 weeks, night sweats, measured weight loss <10% in preceding 6 months); low priority = no TB symptoms. ^3^ Samples tested with Xpert MTB/RIF at the end of the study. ^4^ High priority (any of: current cough, fever ≥ 3 weeks, night sweats ≥ 4 weeks, body mass index (BMI) <18.5 kg/m^2^, CD4 <100x10^6^/l, measured weight loss ≥10% in preceding 6 months, or other feature raising high clinical suspicion of TB).

This “LAM” study was part of XPHACTOR, a prospective cohort study evaluating a risk-based algorithm to prioritise Xpert MTB/RIF testing amongst adults attending for routine HIV care in South Africa. Fig 1 depicts XPHACTOR study flow and how participants entered the LAM substudy.

### XPHACTOR study population and recruitment

We enrolled a systematic sample of adults (aged ≥18 years) attending four clinics in Gauteng province for HIV care, irrespective of presence of symptoms suggestive of TB. Patients taking anti-tuberculosis treatment within the previous 3 months were excluded. Patients were enrolled into “on ART” (currently taking ART) and “pre-ART” (in HIV care but not taking ART) groups. At the time of the study, ART eligibility comprised CD4 ≤350x10^6^/l or WHO clinical stage ≥3.

### XPHACTOR procedures

#### Enrolment

At enrolment, research staff administered a standardised questionnaire incorporating the WHO TB screening tool (any of current cough, fever, night sweats or unintentional weight loss), measured height and weight, and recorded most recent clinic CD4 cell count. Further investigation was prioritised according to the XPHACTOR algorithm with an immediate spot sputum sample sent for Xpert MTB/RIF for (i) all assigned “high priority” (any of: current cough, fever ≥ 3 weeks, body mass index (BMI) <18.5 kg/m^2^, CD4 <100x10^6^/l, measured weight loss ≥10% in preceding 6 months, or other feature raising high clinical suspicion of TB); (ii) those in the pre-ART group with CD4<200x10^6^/l at enrolment because of *a priori* high risk of active TB. For all other participants a spot sputum sample was frozen at -80°C within 24 hours for testing with Xpert MTB/RIF at the end of the study.

#### Follow-up

Participants were reviewed monthly to three months, with repeat WHO symptom screen and a spot sputum requested for Xpert MTB/RIF if “high priority” by the study algorithm at that visit. Those in the “on ART” group who were asymptomatic at enrolment were telephoned at 1 and 2 months to update locator information but were not asked about TB symptoms. At the 3-month visit, sputum (induced if necessary) and blood were collected for mycobacterial culture in liquid media (Bactec MGIT 960 or 9240 systems) from all study participants. We allowed the 3-month visit to be undertaken more than three months post-enrolment in order to maximise study follow up.

Participants who submitted an Xpert sample were reviewed and if Xpert-positive, TB treatment was initiated; if negative, further investigation in accordance with national guidelines was facilitated (chest radiograph, sputum culture and trial of antibiotics).

Clinic medical records were reviewed at the end of the study to ascertain any additional TB diagnoses. We recorded deaths through reports from participant-nominated contacts, clinic staff, and by accessing the Department of Home Affairs vital statistics database using participants’ South African identification (ID) numbers, which enabled us to track vital status several months after final study visit for those with valid ID numbers.

### LAM substudy procedures

All participants with CD4<200x10^6^/l were eligible for this substudy. Eligible participants were asked to provide a spot urine sample in a sterile container at enrolment, which was stored at 2–8°C prior to freezing at -80°C within 24 hours of collection. At the end of the study samples were thawed to ambient temperature and tested with LF-LAM by two trained laboratory technologists in accordance with training provided by Alere representatives. The technologists did not have access to other bacteriological results when performing LF-LAM tests. Each test was graded once, using the pre-January 2014 manufacturer’s reference card comprising five grades of colour intensity with the least intense band assigned grade 1, absence of a band graded negative, and absence of control band deemed a failed test. [12]

### TB Case Definitions

“Confirmed” TB was defined as a positive result on i) Xpert MTB/RIF or ii) line probe assay (LPA) performed on cultured isolate for identification and susceptibility or iii) *M*. *tuberculosis* (*Mtb*) culture, from any sample taken within six months of enrolment. Individuals who started TB treatment within six months of enrolment, in the absence of microbiological confirmation (including those with treatment starts reported at verbal autopsy) and those smear-positive in the absence of an associated positive culture or LPA result, were assigned “clinical” TB. This was based on the assumption that an HIV-positive adult with a positive test result or starting TB treatment within 6 months after enrolment likely had active TB at enrolment, supported by data from Zimbabwe which estimated the mean duration of smear-positivity prior to TB diagnosis amongst HIV-positive adults to be 18–33 weeks. [13] Furthermore individuals diagnosed with clinical TB based on findings at the 3-month visit would only have started treatment after the 3-month visit.

“Not TB” was defined as fulfilling all of the following: absence of criteria for confirmed or clinical TB; alive at least 3 months after enrolment; and ≥1 MTB culture or Xpert result from any sample within 6 months of enrolment. Participants who did not fulfil the case definitions for TB or “not TB” were deemed “unclassifiable” and excluded from the main analysis.

### Statistical methods

Data were analysed using Stata 14 (Stata Corporation, College Station, TX, USA).

We did not undertake a formal sample size calculation for the LAM substudy as the sample size was all those eligible from the parent study. The target sample size for XPHACTOR was based on estimating the sensitivity of the study algorithm, the main aim of the study, with reasonable precision.

We calculated sensitivity, specificity and predictive values with 95% confidence intervals (CI) for LF-LAM using cut-offs of grade ≥1+ and ≥2+ to define LAM-positive against a diagnostic reference standard of i) confirmed plus clinical TB, and ii) confirmed TB with clinical TB excluded from numerator and denominator. We also calculated these parameters for grade ≥1+ cut-off for subgroups stratified by CD4<100x10^6^/l and CD4 ≥100x10^6^/l.

We undertook an exploratory assessment of mortality at six months in all participants who provided a urine specimen, assuming all without record of demise were alive: i) six months after enrolment if they had valid South African ID number, or ii) at latest study / clinic visit date (hereafter last visit) if no valid South African ID. Person-time was calculated from the date of study enrolment until: date of death if death recorded within six months of enrolment, and for all others six months from enrolment if valid South African ID or date of last visit if no valid South African ID. We constructed Kaplan-Meier curves of survival probability by LAM positivity using LAM grade ≥1+ to define positivity, and compared mortality using Cox regression.

### Ethics statement

The study was approved by the ethics committees at the University of the Witwatersrand, University of Cape Town, and the London School of Hygiene & Tropical Medicine. All participants gave written informed consent, or witnessed verbal consent if unable to write. For illiterate participants, an impartial witness was present during the consenting process, and signed the witness section of the consent form. All ethics committees approved this consent procedure. Consent and participation in the study was voluntary. Participants were able to refuse to take part, with no consequences to their healthcare or any other services as a result of refusal.

## Results

**Fig 2:**
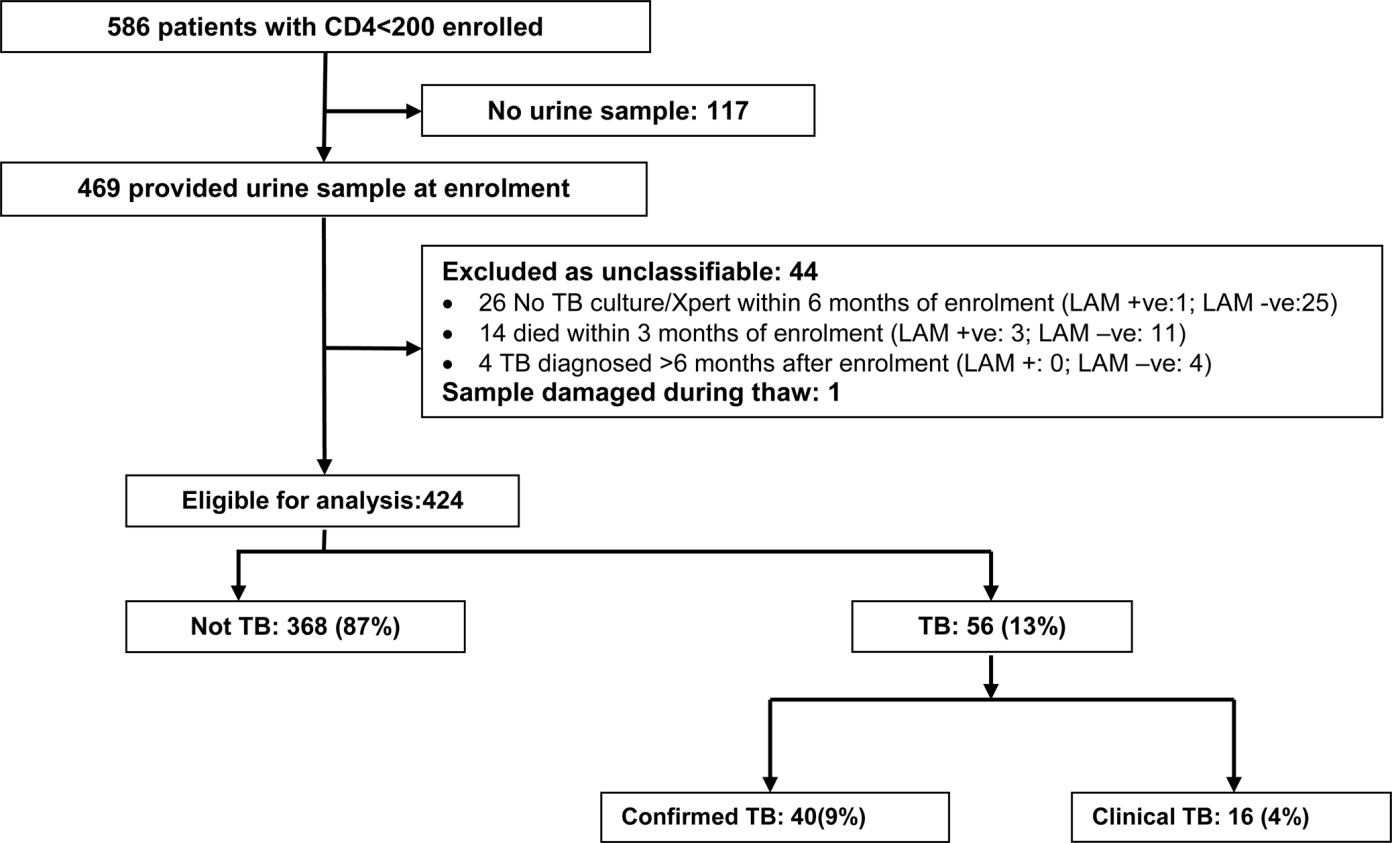
Flow chart of study participants. LAM+ defined as ≥ grade 1.

Between September 2012 and March 2014 we enrolled 3508 participants established in HIV care, of whom 586 had CD4 <200x10^6^/l and were eligible for the LAM substudy. 80% (469/586) provided a urine sample, and the remaining 20% (117/586) did not, as unable (93) or reason not recorded (24) (Fig 2); 67% (395/586) provided a spot sputum sample at enrolment. 44 participants were excluded because unclassifiable: no TB diagnosed but absence of any TB microbiology results (26); death within three months of enrolment (14); and TB diagnosed >6 months after enrolment (4); and one sample could not be tested as damaged, leaving 424 eligible for evaluation of diagnostic accuracy of LF-LAM.

There was little difference in WHO-tool positivity or gender amongst those providing (N = 469) vs. not providing urine (N = 117): 52% vs. 44% WHO-tool positive (p = 0.1) and 61% vs. 62% female (p = 0.7). Median CD4 was lower in those providing urine compared with those who did not (110 vs. 140x10^6^/l [p = 0.01]); and a greater proportion also provided sputum at enrolment (74% vs. 42% [p<0.001]).

### Participant characteristics

Characteristics of the 424 LAM substudy participants are presented in Table 1. The majority of participants were female (61%), WHO-tool positive at enrolment (53%), and in the “on ART” group (57%) amongst whom median duration of ART was 22 months (interquartile range [IQR] 6, 52). Median age, CD4 and BMI at enrolment were respectively 39 years, 111x10^6^/l, and 23 kg/m^2^; and 30% had previously been treated for TB. In the “pre-ART” group, for 97% (176/182) participants with reported date of first positive HIV test recorded, median duration since HIV diagnosis was 20 days (IQR 10, 65). 94% (171/182) of “pre-ART” initiated ART within median 9 days (IQR 4, 24) from enrolment (initiation date available for 170/171).

**Table 1:** Baseline characteristics of study participants.

	All participants N = 424
**Age-years**	
Median (IQR)	39 (32, 45)
**Sex**	
Female	258 (60.8%)
**Ethnic group**	
Black/African	421 (99.3%)
**Participant category**	
Pre-ART	182 (42.9%)
On ART	242 (57.1%)
ART duration for those on ART, months median (IQR)	22 (6, 52)
**Previous TB treatment**	
Yes	125 (29.5%)
**Ever had IPT**	
Yes	18 (4.2%)
**Ever had CPT**	
Yes	276 (65.1%)
**Current diuretic use**	
Yes	10 (2.4%)
**Enrolment WHO symptom screen**	
Positive	224 (52.8%)
**Enrolment BMI-kg/m**^**2**^ **(N = 423)**	
Median (IQR)	23 (20, 27)
**Enrolment CD4 x10**^**6**^**/l** ^**1**^	
Median (IQR)	111 (56, 161)

Numbers are median (Interquartile range [IQR]) or number (%)

ART = Antiretroviral therapy; CPT = Co-trimoxazole preventive therapy; IPT = Isoniazid preventive therapy; BMI = body mass index

WHO symptom screen positive = self-report of any of current cough, fever, night sweats or unintentional weight loss

^1^ All participants had CD4<200x10^6^/l

The proportion reporting each WHO-tool symptom was cough, 32% (134/424) with median duration 14 days (interquartile range [IQR] 7,38); unintentional weight loss, 31% (131/424); night sweats, 15% (65/424); fever, 10% (44/424); and 24% (102/424) reported more than one symptom.

### Tuberculosis diagnoses

13% (56/424) of participants fulfilled our case definitions for tuberculosis (7% [16/242] “on ART” and 22% [40/182] “pre-ART”), amongst whom treatment start date was available for 53/56, and was started at a median of 13 (IQR 5, 95) days from enrolment. 40/56 had confirmed TB (25 Xpert-positive, 7 Mtb culture-positive, 7 both Xpert and Mtb culture-positive, 1 pleural fluid cultured isolate LPA-positive) of whom 36/40 had pulmonary TB, 3/40 both pulmonary and extrapulmonary TB, and 1/40 extrapulmonary TB only. 16/56 had clinical TB for whom diagnosis was based on compatible chest radiograph or abdominal ultrasound (8), persistent cough and weight loss (1), positive sputum smear (1), and unknown (6) including TB treatment reported at verbal autopsy (2). Amongst those with clinical TB the site was pulmonary (10/16), extrapulmonary (4/16), and not recorded (2/16).

### Performance of urine LAM

LAM results were available for 424 participants. A positive LF-LAM result using grade 1 vs. grade 2 cut-off was observed in 5.7% (24) vs. 1.9% (8) of participants. The distribution of results was negative, 94% (400); grade 1, 3.8% (16); grade 2, 1.2% (5); grade 3, 0.5% (2); and grade 5, 0.2% (1).

Table 2 summarises the performance of LF-LAM in our study population. Sensitivity for all TB (clinical and confirmed) using grade 1 cut-off was 14.3% (95% CI 6.4%, 26.2%), similar if reference standard was confirmed TB with all clinical TB excluded (12.5% [95% CI 4.2%, 26.8%]), but lower if grade 2 cut-off utilised (5.4% [95% CI 1.1%, 14.9%] for all TB). Sensitivity was greater in participants with enrolment CD4<100 vs. CD4 ≥100x10^6^/l: using grade 1 cut-off 17.1% (95% CI 6.6%, 33.6%) vs. 9.5% (95% CI 1.2%, 30.4%) for all TB, and 16.7% (95% CI 4.7%, 37.4%) vs. 6.3% (95% CI 0.2%, 30.2%) for confirmed TB. Specificity of the test was >95% irrespective of reference standard, CD4 stratum, or whether positivity was defined using grade 1 or grade 2 cut-off.

**Table 2:** Diagnostic accuracy of LF-LAM among HIV clinic attendees with CD4<200.

	Prevalence of TB		Prevalence of positive LAM		Sensitivity		Specificity		PPV		NPV	
Gold standard = confirmed* and clinical^†^ TB	**n/N**	**%**	**n/N**	**%**	**n/N**	**% (95% CI)**	**n/N**	**% (95% CI)**	**n/N**	**% (95% CI)**	**n/N**	**% (95% CI)**
***Grade 1***^***‡***^ ***cut-off***	56/424	13.2%	24/424	5.7%	8/56	14.3% (6.4, 26.2)	352/368	95.7% (93.0, 97.5)	8/24	33.3% (15.6, 55.3)	352/400	88.0% (84.4, 91.0)
CD4 <100	35/187	18.7%	12/187	6.4%	6/35	17.1% (6.6, 33.6)	146/152	96.1% (91.6, 98.5)	6/12	50.0% (21.1, 78.9)	146/175	83.4% (77.1, 88.6)
CD4 ≥100	21/237	8.9%	12/237	5.1%	2/21	9.5% (1.2, 30.4)	206/216	95.4% (91.7, 97.8)	2/12	16.7% (2.1, 48.4)	206/225	91.6% (87.1, 94.8)
***Grade 2***^***§***^ ***cut-off***	56/424	13.2%	8/424	1.9%	3/56	5.4% (1.1, 14.9)	363/368	98.6% (96.9, 99.6)	3/8	37.5% (8.5, 75.5)	363/416	87.3% (83.7, 90.3)
Gold standard = confirmed* TB (all clinical^**†**^ **TB excluded) (N = 408)**												
***Grade 1***^***‡***^ ***cut-off***	40/408	9.8%	21/408	5.1%	5/40	12.5% (4.2, 26.8)	352/368	95.7% (93.0, 97.5)	5/21	23.8% (8.2, 47.2)	352/387	91.0% (87.6, 93.6)
CD4 <100	24/176	13.6%	10/176	5.7%	4/24	16.7% (4.7, 37.4)	146/152	96.1% (91.6, 98.5)	4/10	40.0% (12.2, 73.8)	146/166	88.0% (82.0, 92.5)
CD4 ≥100	16/232	6.9%	11/232	4.7%	1/16	6.3% (0.2, 30.2)	206/216	95.4% (91.7, 97.8)	1/11	9.1% (0.2, 41.3)	206/221	93.2% (89.1, 96.2)
***Grade 2***^***§***^ ***cut-off***	40/408	9.8%	8/408	2.0%	3/40	7.5% (1.6, 20.4)	363/368	98.6% (96.9, 99.6)	3/8	37.5% (8.5, 75.5)	363/400	90.8% (87.5, 93.4)

* Confirmed TB = positive on Xpert MTB/RIF or line probe assay or M. tuberculosis culture, from any sample taken within 6 months of enrolment

† Clinical TB = started TB treatment within 6 months of enrolment, in the absence of microbiological confirmation and those smear-positive in the absence of an associated culture

‡ Grade 1 positive: > = 1+

§ Grade 2 positive: > = 2+

NPV = Negative predictive value; PPV = Positive predictive value; CI = confidence interval

In a sensitivity analysis we included the 40 participants whom we deemed to have unclassifiable TB outcome, and considered those excluded because deceased within 3 months of enrolment to have TB (N = 14), and those excluded because of absence of microbiology to not have TB (N = 26). Against a reference standard for all TB (clinical and confirmed) and using grade 1 cut-off, the sensitivity and specificity of LF-LAM was 15.7% (95% CI 8.1, 26.4) and 95.7% (95% CI 93.2, 97.5) respectively. If a grade 2 cut-off was used the sensitivity and specificity of LF-LAM was 8.6% (95% CI 3.2, 17.7) and 98.7% (95% CI 97.1, 99.6) respectively.

There were five false positive LF-LAM rests using the grade 2 cut-off, of these one participant with CD4 of 4x10^6^/l at enrolment had *M*. *avium* isolated from sputum culture but was not treated and was alive at six months. All of the remaining four participants with false positive LF-LAM had negative sputum mycobacteriology during follow up and were alive at six months.

### Mortality

**Fig 3:**
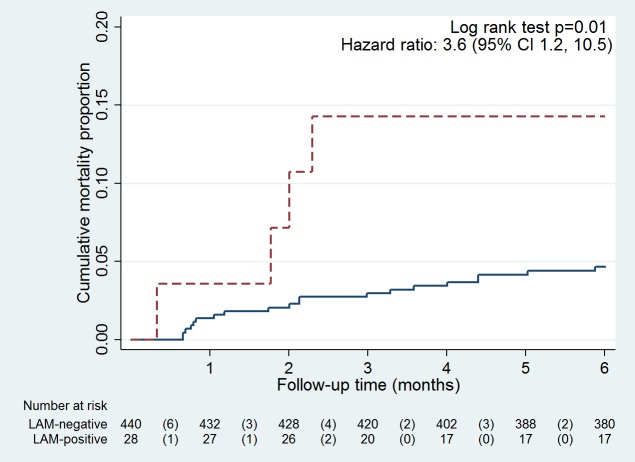
Kaplan-Meier curve comparing mortality between LAM positive (dashed line) and LAM negative (solid line) participants using grade 1 cut-off. Y-axis range for cumulative mortality is 0 to 0.2

Amongst 468 participants with evaluable urine samples, 6% (28/468) were LF-LAM positive using grade 1 cut-off, of whom 14% (4/28) died within six months of enrolment. Among the 440 who were LF-LAM negative, 5% (20/440) died (hazard ratio 3.6 [95%CI 1.2, 10.5], p = 0.04; Fig 3).

## Discussion

We found very low sensitivity of LF-LAM for TB (whether confirmed, or also including clinical diagnoses) among ambulatory outpatients established in HIV care with CD4<200x10^6^/l, which does not support its use for TB screening in this study population. Recent WHO LF-LAM policy guidance recommends against use for TB screening, based on the systematic review to which our data contributed, which reported (using grade 2 cut-off and microbiological reference standard) a pooled sensitivity of only 23% for screening HIV-positive outpatients.[11] Our study is the first reporting performance of LF-LAM among outpatients established in HIV care. It adds to the published evaluations of LF-LAM amongst HIV-positive adult outpatients screened for TB, which to date have only been undertaken in those newly diagnosed [6, 7] or about to initiate ART, [4, 5] populations likely to be sicker than those already in HIV care.

In our study, LF-LAM had a sensitivity of 13% using the grade ≥1 cut-off (against confirmed TB as a gold standard) vs. reported 26–29% [4, 5] in those about to initiate ART, and 28–41% [6, 7] amongst those with a new HIV diagnosis. In those evaluations, sensitivity improved greatly at lower CD4 counts, e.g. for confirmed TB from around 20% if CD4≥100x10^6^/l to >50% if CD4 <100x10^6^/l; our improvement from 10% to 17% is in accord although still not useful for screening. [4–7] The sensitivity of LF-LAM is greater in those who are sicker, and this test is most useful as a rapid rule-in tool for TB when used for HIV-positive inpatients with symptoms suggestive of TB and advanced immunosuppression, a population with high mortality; and its use in this setting is supported by the recent WHO guidance.[11] In these populations sensitivity of 59–66% [9, 10] for culture-confirmed TB has been reported from evaluations in South Africa and Uganda, increasing to 85% [10] when combined with sputum Xpert MTB/RIF, thus potentially enabling rapid initiation of TB treatment. In contrast our participants were established in HIV care, and we were evaluating the usefulness of LF-LAM as part of a routine screening algorithm for intensified TB case finding in HIV clinic attendees, although restricted to those with CD4 counts below 200x10^6^/l for whom LF-LAM was most likely to be useful. The median CD4 in our study population was 111x10^6^/l which is lower than the 170–248x10^6^/l reported in outpatient evaluations, [4–7] and our participants were frequently symptomatic, but in spite of this we found poor sensitivity for TB, possibly as patients were less ill.

An obvious advantage of a urine-based diagnostic test is ease of specimen collection, although privacy is clearly required. However, we found 20% of eligible patients did not provide a urine sample which, although less than the 33% unable to produce sputum spontaneously at enrolment, is still greater than 1–3% unable to produce urine in other outpatient studies. [5, 7] We found no indication that those unable to produce urine were more unwell than those who could, so this is unlikely to have affected our findings, although we acknowledge this as a limitation of our study. Our study procedures were fitted around routine appointments in busy public sector clinics, and we postulate that time limitations may have contributed, as these patients were also less likely to provide sputum at enrolment.

We found increased mortality amongst patients who were LF-LAM positive, consistent with studies of sicker inpatient HIV-positive populations reporting LAM-positivity as a predictor for mortality. [14, 15]

Strengths of our study include our systematic sampling and longitudinal follow-up, which minimised the number of TB diagnoses missed. All samples for mycobacteriology collected during the course of our study, many of which were sputum samples collected at enrolment, contributed to our reference standard of “confirmed” TB. We undertook all LF-LAM tests at study completion using frozen samples, but this is consistent with other published studies, and all our samples were stored in accordance with manufacturer’s recommendation with only one freeze-thaw cycle, and processed within one year from collection. [16] A limitation of our study is the exclusion of participants (N = 40) who were not diagnosed with TB but either did not have any TB microbiology results or died within three months of enrolment, but our sensitivity analysis shows that this made little difference to the performance of LF-LAM.

## Conclusion

Despite the appeal of LF-LAM as a cheap, non-sputum based, point-of-care TB screening tool, the low sensitivity in this population with advanced immunosuppression, of whom 13% had TB, precludes recommendation for its use to screen for TB in ambulatory patients established in HIV care in accordance with the recent WHO LF-LAM policy guidance.[11]
